# Alterations in auditory brain stem response distinguish occasional and constant tinnitus

**DOI:** 10.1172/JCI155094

**Published:** 2022-03-01

**Authors:** Niklas K. Edvall, Golbarg Mehraei, Martin Claeson, Andra Lazar, Jan Bulla, Constanze Leineweber, Inger Uhlén, Barbara Canlon, Christopher R. Cederroth

**Affiliations:** 1 Laboratory of Experimental Audiology, Department of Physiology and Pharmacology, Karolinska Institutet, Stockholm, Sweden.; 2 Decibel Therapeutics Inc., Boston, Massachusetts, USA.; 3 Stress Research Institute (Stressforksningsinsitutet), Stockholm University, Stockholm, Sweden.; 4 Hörsel-och Balanskliniken, Karolinska Universitetssjukhuset, Stockholm, Sweden.; 5 University of Bergen, Bergen, Norway.; 6 Department of Psychiatry and Psychotherapy, University of Regensburg, Regensburg, Germany.; 7 National Institute for Health Research (NIHR) Nottingham Biomedical Research Centre, Nottingham University Hospitals NHS Trust, Nottingham, United Kingdom (UK).; 8 Hearing Sciences, Division of Clinical Neuroscience, School of Medicine, University of Nottingham, Nottingham, UK.

**Keywords:** Neuroscience, Otology, Epidemiology, Neurological disorders

## Abstract

**BACKGROUND:**

The heterogeneity of tinnitus is thought to underlie the lack of objective diagnostic measures.

**METHODS:**

Longitudinal data from 20,349 participants of the Swedish Longitudinal Occupational Survey of Health (SLOSH) cohort from 2008 to 2018 were used to understand the dynamics of transition between occasional and constant tinnitus. The second part of the study included electrophysiological data from 405 participants of the Swedish Tinnitus Outreach Project (STOP) cohort.

**RESULTS:**

We determined that with increasing frequency of the occasional perception of self-reported tinnitus, the odds of reporting constant tinnitus after 2 years increases from 5.62 (95% CI, 4.83–6.55) for previous tinnitus (sometimes) to 29.74 (4.82–6.55) for previous tinnitus (often). When previous tinnitus was reported to be constant, the odds of reporting it as constant after 2 years rose to 603.02 (524.74–692.98), suggesting that once transitioned to constant tinnitus, the likelihood of tinnitus to persist was much greater. Auditory brain stem responses (ABRs) from subjects reporting nontinnitus (controls), occasional tinnitus, and constant tinnitus show that wave V latency increased in constant tinnitus when compared with occasional tinnitus or nontinnitus. The ABR from occasional tinnitus was indistinguishable from that of the nontinnitus controls.

**CONCLUSIONS:**

Our results support the hypothesis that the transition from occasional to constant tinnitus is accompanied by neuronal changes in the midbrain leading to a persisting tinnitus, which is then less likely to remit.

**FUNDING:**

This study was supported by the GENDER-Net Co-Plus Fund (GNP-182), the European Union’s Horizon 2020 grants no. 848261 (Unification of Treatments and Interventions for Tinnitus [UNITI]) and no. 722046 (European School for Interdisciplinary Tinnitus Research [ESIT]).

## Graphical abstract

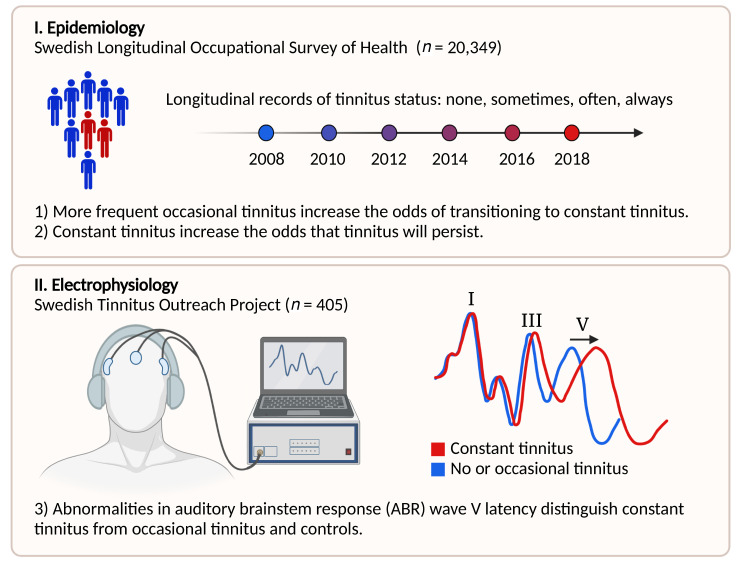

## Introduction

Tinnitus is the perception of sounds in the absence of any external sound source. It is a common condition that is experienced by nearly 15% of the population and has been shown to be influenced by not only environmental factors, but also genetics (1–6). For those with severe tinnitus (1 out of 10), the significant impact on life quality is accompanied by a high clinically unmet need (7). A major hurdle in the development of effective strategies for treating tinnitus is the lack of objective measures that can be used to assess treatment outcomes (8). In spite of emerging reports showing the involvement of limbic structures in the affective components of tinnitus (9–11), a recent systematic review reports that there is no evidence for a reliable and reproducible objective measure that quantifies tinnitus (12).

The current pathophysiological model suggests that tinnitus arises as a maladaptive plasticity in response to diminished sensory input, whereby damage to the ear leads to changes in homeostatic gain control in the auditory brain stem and the auditory cortex (e.g., increased spontaneous firing rate, increased neural synchrony, increased gain) as well as thalamocortical dysrhythmia (for review, see ref. 13). Thus, electrophysiological measures could potentially provide means to detect neurological alterations occurring in tinnitus patients. The auditory brain stem response (ABR) has been suggested as a tool for measuring such alterations in response to sound stimuli in spite of tinnitus not being elicited by sounds. An ABR is an evoked auditory potential whose output consists of 5 waves sequentially corresponding to the auditory nerve (AN), the cochlear nucleus (CN), the superior olivary complex (SOC), the lateral leminiscus (LL), and the inferior colliculus (IC), where the amplitude reflects the number of neurons firing and the latency is determined by the synchrony and speed of neural transmission. However, studies assessing the use of ABRs for the diagnosis of tinnitus show mixed results, likely as a result of poor tinnitus definitions and low sample size (14). For instance, Schaette and McAlpine suggest that ABR wave I amplitude, inferring for hidden hearing loss, is associated with tinnitus (15); however, these findings were not consistently replicated (16–18) and remain a matter of debate (14). With regard to wave V latency, the same issues of replication persist whereby nearly half of the studies show delayed wave V latencies in tinnitus subjects, but not the other half (14). The aim of this study is to address these controversies using a large cohort of deeply phenotyped tinnitus and control subjects and provide additional insights on the dynamics of the transition between occasional and constant tinnitus.

## Results

### Sociodemographics of the SLOSH cohort.

We first studied the relationship between occasional and constant tinnitus in a large cohort in Sweden, the Swedish Longitudinal Occupational Survey of Health (SLOSH). From 2008 to 2018, data on tinnitus were collected every 2 years from a total of 20,439 participants, adding up to 53,273 observations. The prevalence of occasional or constant tinnitus at each follow-up with respect to the tinnitus status reported in the previous data collection is shown in Supplemental Table 1 and includes sociodemographics (age, sex, and education) (supplemental material available online with this article; https://doi.org/10.1172/JCI155094DS1). Generally, men, older participants, and those with lower education reported constant tinnitus more often.

Supplemental Table 2 describes all the combinations of transitions into various tinnitus states from one data collection to the following one after 2 years and illustrates the high instability of the occasional group. Interestingly, a greater proportion of individuals was found transiting toward constant tinnitus as the tinnitus perception in the previous data collection increased in frequency (sometimes < often < constant). Only 0.8% changed from reporting sometimes to constant tinnitus, but 1.21% changed from having often to constant tinnitus. However, 7% who reported constant tinnitus 2 years before also did so at the next follow-up.

### Dynamic progression from occasional to constant tinnitus.

To provide estimates of these dynamics, we applied the generalized estimating equation (GEE) method. The results can be found in Supplemental Table 3. Estimates based on an unstructured correlation structure (model 1) or an exchangeable correlation structure (model 2) produced highly comparable results. Thus, we will continue by presenting the results of model 1. The GEE analysis confirmed that the risk of constant tinnitus is greater with the increasing frequency of occasional tinnitus (previous tinnitus [some]: adjusted odds ratio [aOR] and 95% CI = 5.62 [4.83–6.55], *P* <.0001; previous tinnitus [often] aOR: 29.74 [25.69–34.42], *P* <.0001; Supplemental Table 3). Interestingly, the risk of having constant tinnitus at a 2-year follow-up when already having constant tinnitus was very high (previous tinnitus [constant] aOR: 603.02 [524.74–692.98], *P* <.0001), suggesting that once constant, the likelihood of persisting in this state was high. Hence, the progression toward constant tinnitus appears to be related to the frequency with which tinnitus is perceived. As the analysis revealed a significant effect of sex (*P* <.0001), we also performed sex-stratified analyses (Supplemental Tables 4–5). These did not reveal any sex bias in any of the associations.

### Characteristics of the STOP participants.

**Figure 1:**
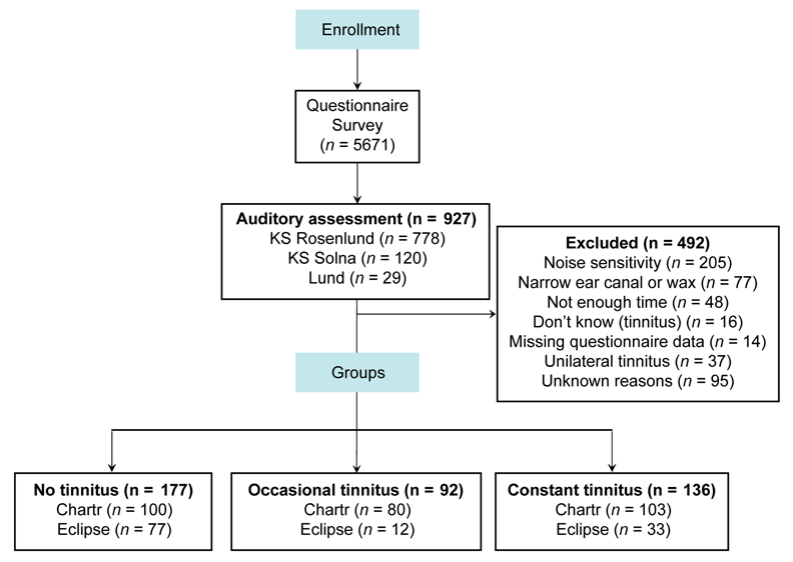
Participant flow diagram. Overview of participants excluded and recruited for the electrophysiological study.

**Figure 2:**
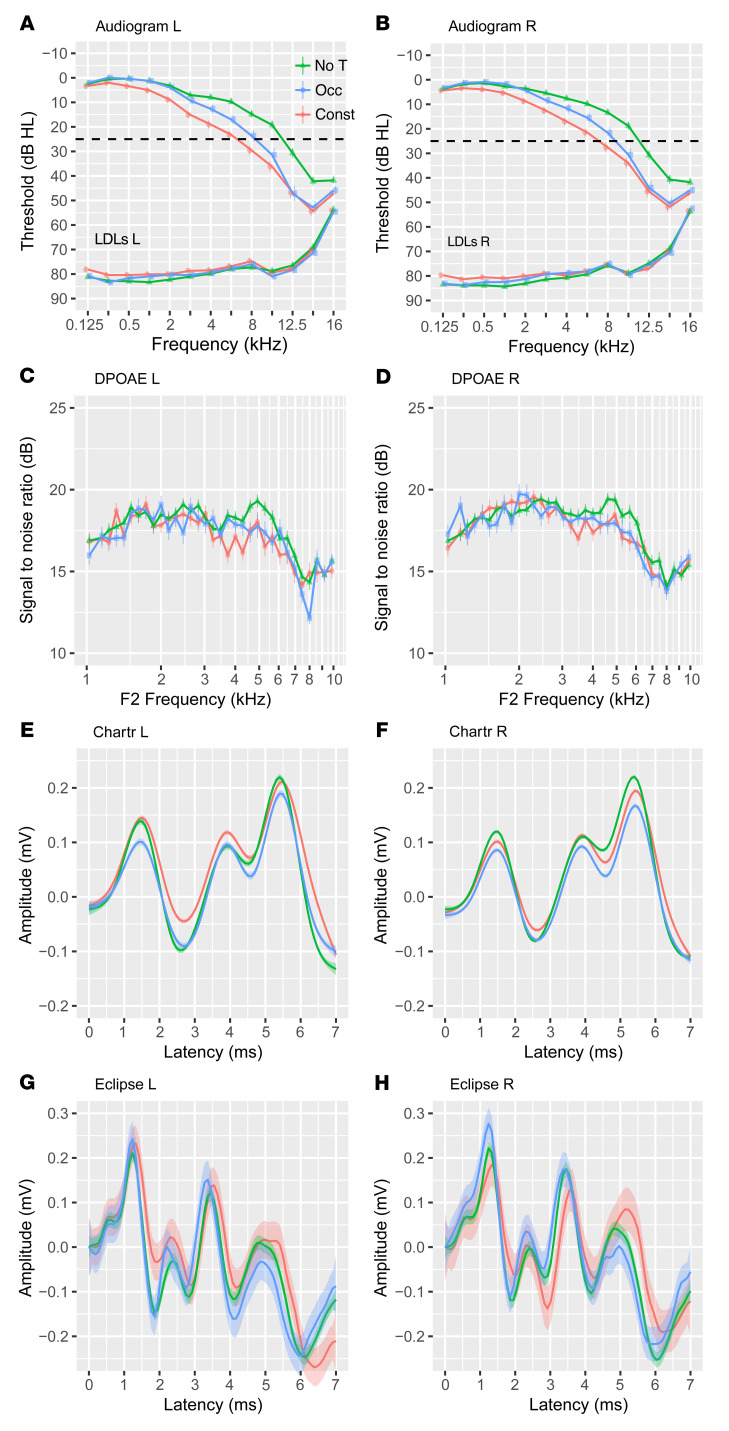
Auditory measures from no tinnitus control, occasional tinnitus, and constant tinnitus. Pure tone audiograms: hearing thresholds (top line) and LDLs (bottom line) from left (**A**) and right (**B**) ears of nontinnitus controls(green triangles; *n* = 177), individuals with occasional tinnitus (blue squares; *n* = 92), and those with constant tinnitus (red circles; *n* = 136) from 0.125 to 16 kHz. Results are represented as mean values ± SEM. Symbols are made with slight transparency to not obscure error bars. DPOAEs from left (**C**) and right (**D**) ears, signal-to-noise ratio of the first distortion product displayed as a function of the F2 frequency (kHz). Labels are the same as in panels **A** and **B**. No tinnitus, *n* = 177; occasional tinnitus, *n* = 92; constant tinnitus, *n* = 110. Statistics obtained from clinically standardized PTA 4 and PTA HF (high frequency) as well as DPOAEs are shown in Table 2. (**E** and **F**) ABRs acquired with the Chartr: no tinnitus (*n* = 100), occasional tinnitus (*n* = 73), constant tinnitus (*n* = 99). (**G** and **H**) ABRs acquired with the Eclipse: no tinnitus (*n* = 77), occasional tinnitus (*n* = 12), constant tinnitus (*n* = 32) for left (**E** and **G**) and right (**F** and **H**) ears separately. No T, no tinnitus controls (green line); Occ, occasional tinnitus (blue line); Const, constant tinnitus (red line). Results are represented as mean values ± 95% CIs. Corresponding statistics are shown in Supplemental Table 6. Note: incomplete ABR wave forms were excluded in the graphs.

We next hypothesized that this robust transition toward constant tinnitus could be reflected by changes in neurological responses. We thus assessed ABRs from individuals with constant and occasional tinnitus as well as nontinnitus controls from the Swedish Tinnitus Outreach Project (STOP) (Figure 1). The sociodemographics of the 3 groups are presented in Table 1, along with measures of psychological and life-quality impact, conventionally assessed in tinnitus studies (19, 20). We also assessed tinnitus-related comorbidities known to be strongly associated with tinnitus (21–23) as well as auditory features (Table 2). The participants with tinnitus more commonly reported having hearing difficulties (*P* < 0.001) and using devices to help with hearing (*P* < 0.001), consistent with greater hearing loss in both the left and right ears (see audiograms in Figure 2, A and B, and the average PTA 4 [defined as pure tone average at 0.5, 1, 2, and 4 kHz] and PTA HF [defined as pure tone average at 10, 12.5, 14, and 16 kHz] reported in Table 2). The loudness discomfort levels (LDLs) appeared as significantly different across the groups (Table 2, *P <* 0.001) for the same 4 frequency average, albeit with minimal clinical relevance (Figure 2, A and B). Moreover, LDL values at 14 and 16 kHz marked an “unreached threshold” for most of the participants, as hardware limitations prevented the presentation at high enough levels. In contrast, the hyperacusis questionnaire (HQ) revealed a marked increase in sound intolerance in the tinnitus participants (Table 1). The distortion products of otoacoustic emissions (DPOAEs) were less regular (Figure 2, C and D) and only differed among the groups in the left ear (Table 2). Consistent with previous studies (16, 24), the speech-in-noise scores were also found to be lower in individuals with tinnitus (Table 2).

### Wave V of the ABR distinguishes constant from occasional tinnitus.

The ABR wave forms are shown in Figure 2, E–H, for the 2 hardware systems used (Chartr EP from Otometrics and Eclipse from Interacoustics), both commonly used in clinics. These two systems, however, differed in their amplitude and latency output, likely because of the differences in transducers, which is illustrated with the peak latency of the ABR wave I (Supplemental Table 6). For instance, the average ABR wave I amplitude differed from 0.03 up to 0.13 μV in constant tinnitus when compared with the nontinnitus controls, and the ABR wave V latencies increased from 0.18 up to 0.20 ms on the Chartr and the Eclipse, respectively. The results of the univariate logistic regression are shown in Supplemental Figures 1–3. Since the ABRs’ amplitudes and latencies are highly confounded by age, sex, hearing, hyperacusis, and hardware, we constructed a stepwise-selected logistical regression model controlling for all of these variables. Since the LDLs showed limitations in being determined at high frequencies (>12.5 kHz), we instead used the HQ, which has excellent reliability (25–27), to control for hyperacusis.

In the models comparing the control group with those with occasional tinnitus, no ABR variables were selected in a stepwise-selected model, indicating that occasional tinnitus and nontinnitus cannot be distinguished using ABR. However, when comparing the control group with those with constant tinnitus, 5 ABR features were included in their respective selected model. Left ear wave III and V latency — together with age, HQ score, and the hearing variables PTA 4 and PTA HF — showed ORs of 12.27 (95% CI: 3.14–52.19, *P* = 0.001) and 4.31 (1.85–10.53, *P* = 0.002), respectively (Table 3). Comparing the same 2 groups, the right ear ABR variables again showed wave III latency as a significant variable, with an OR of 10.44 (2.52–45.73, *P* = 0.002). The amplitude measurements for waves III and V were also included after stepwise selection, with ORs of 0.03 (0.004–0.21, *P* = 0.001) and 0.07 (0.008–0.55, *P* = 0.013), respectively (Table 3). In models for occasional and constant tinnitus, the only ABR variables that were included after selection were wave I and V latency for left ears with ORs of 41.75 (2.50–848.43, *P* = 0.011) and 7.21 (2.51–22.34, *P <* 0.001), respectively. Such a large interval in the OR could make one question the quality of the data, but the test-retests showed good to excellent reliability for ABR latencies in both systems, while ABR amplitude had generally poor reliability, with the exception of the wave I amplitude response obtained with the Eclipse, which was excellent (Supplemental Table 7). Thus, the variability when comparing constant and occasional tinnitus resides more in the biology of the latter, which is more “unstable,” with a greater occurrence of recovery and relapses than constant tinnitus, as illustrated by the epidemiological data (Supplemental Table 2). In conclusion, wave V latency for left ears distinguishes constant tinnitus from occasional tinnitus and nontinnitus controls.

## Discussion

Tinnitus is a heterogeneous condition, and objective measures that could segregate tinnitus subgroups would not only optimize the classification of tinnitus patients, but also improve the assessment of therapeutic outcomes (28, 29). This work provides an assessment of ABR changes in tinnitus subjects, distinguishing those with occasional from those with constant tinnitus as well as comparing the left and right ears. Our findings confirm that constant tinnitus is associated with increased ABR wave V latency when compared with either occasional tinnitus or nontinnitus controls when controlling for biological variables such as age, sex, hearing loss, and hyperacusis. This points toward a dysregulation of neuronal processing in the midbrains of individuals who have progressed from occasional to constant tinnitus, in accordance with the epidemiological data reported here. Our results are consistent with those of smaller studies (*n* = 17–76) reporting delayed wave V of ABRs in tinnitus patients with “permanent annoying tinnitus” (30) or unspecified tinnitus (31) when compared with age-, sex-, and hearing-matched nontinnitus controls. This was also shown for tinnitus subjects with normal hearing thresholds and uni- or bilateral tinnitus (32, 33) as well as subjective, idiopathic tinnitus (32). Wave 5 latency changes were also reported in preclinical studies involving rats displaying behavioral signs of tinnitus 2 to 4 weeks after being exposed to mild noise, causing temporary threshold shifts (34), suggesting that noise exposure could be a cause of midbrain latency changes associated with tinnitus. We previously reported in animals and humans that wave V latency changes could reflect different degrees of AN fiber loss in spite of equal hearing abilities (35), which we propose as an underlying mechanism for the development of constant tinnitus. Of note, interwave latencies could not distinguish any type of tinnitus and the controls. A number of studies, however, failed to show changes in latency in tinnitus patients (14). Reasons for such failure may stem from a low sample size and a poor characterization of tinnitus occurrence of perception (occasional and constant), which may have masked the effects. Indeed, Hofmeier et al. recently showed that hyperacusis could be a strong confounder when assessing ABRs in tinnitus patients: tinnitus individuals without hyperacusis have delayed and reduced ABR wave V, whereas tinnitus individuals with hyperacusis display enhanced ABR wave III and ABR wave V amplitudes for high sound intensities (36). Although we do not stratify for hyperacusis, but instead correct for it, the present study validates these observations in a large cohort of deeply phenotyped tinnitus and control individuals and provides additional insights on the dynamics of the transition between occasional and constant tinnitus. We propose constant tinnitus as a defined subtype, distinguishable from occasional tinnitus by means of electrophysiology.

Interestingly, our results go against the notion of tinnitus being associated with hidden hearing loss (15), which, in animals, is linked to cochlear synaptopathy. Cochlear synaptopathy has been evidenced in mice exposed to mild levels of noise, causing no changes in hearing thresholds, but leading to the decreased wave I amplitude of the ABR and the decreased number of presynaptic ribbons at the inner hair cell–afferent neuron synapse (37). This study was followed up with evidence that low spontaneous-rate fibers were most vulnerable (38), a finding that was further confirmed in nonhuman primates (39). In humans, the association of both synaptic damage and a decreased ABR wave I in normal-hearing individuals is challenging, and thus, suggestive evidence is required to address this indirectly. Whereas postmortem studies have revealed that cochlear synaptopathy occurs in humans (40, 41), its assessment with ABR has been controversial. Some studies have evidenced lower wave I of the ABR in normal-hearing subjects with histories of noise exposure (42–45), while others failed to reveal such findings (46–50). The potential reasons for such conflicting data have been recently debated (51) and include (a) greater variability of the sound-evoked potentials in humans, (b) inadequate sensitivity of the ABR to human synaptopathy, (c) variability in noise exposure in humans, and (d) differences in the control groups across the studies. In the context of tinnitus, Schaette et al. first suggested that tinnitus is associated with lower wave I amplitude in normal-hearing individuals (15). However, a recent systematic review highlighted conflicting results, with some publications reporting effects on wave I amplitude and others failing to do so (14). Milloy et al. suggested that the conflicting results originated from poor tinnitus definitions, low sample size, different distributions of sex across groups, and the use of different transducers across the studies (14). Our study also reveals that different clinical systems have different levels of reliability, from poor to excellent (Supplemental Table 7), in particular for wave I amplitudes, which could explain the discrepancies observed in the literature. This study addresses these limitations in a large sample of the general population using a backward/forward stepwise-selected model based on the Bayesian information criterion (BIC). Consequently, the results presented here are adjusted for the most important factors known to affect ABR wave amplitude and latency — namely, age, sex, hearing, and hyperacusis — allowing us to reveal the genuine relationship between tinnitus and wave V latency changes. Our findings illustrate the importance of controlling for these variables, which becomes clear when comparing the univariate models with more stringent, stepwise-selected ones.

The effects on wave V latency observed here could originate from brain structures even more central than the brain stem, a so-called centrifugal effect from the auditory cortex (52). Similarly, other central components influence tinnitus annoyance, including limbic structures responsible for affective components of tinnitus and pointing to cortical-limbic dysregulation (or frontostriatal gating) in tinnitus (10, 53–55). Given the importance of these networks on tinnitus, the centrifugal influence of the cortex over wave V latencies in tinnitus should be further investigated in the future.

Our study has some limitations. The longitudinal and electrophysiological data were collected from different groups of participants, and thus, longitudinal studies seeking to validate our findings should consider the acquisition of both electrophysiological and survey data simultaneously, with 2 years’ follow-up. The tinnitus participants from STOP presented an average tinnitus handicap inventory (THI) score of 10.81 (±9.78) and 29.72 (±23.89) mean ± SD for the groups reporting occasional and constant tinnitus, respectively. These scores constitute a “negligible” or “light” problem and thus represent a population with less severe tinnitus than what is found in clinical patients (56). These findings may not apply to patients with clinically significant tinnitus. Thus, additional work is required to determine whether our results would be applicable to a clinical sample. Furthermore, recall bias can be strong in subjects with tinnitus. The longitudinal data presented here show that it is not uncommon for participants with occasional tinnitus to switch to another category, which introduces some uncertainty when comparing the ABR results from participants reporting “occasional” tinnitus against the controls, as illustrated with the highly variable ABR data from the participants with occasional tinnitus. However, we confirmed all of the participants’ tinnitus statuses during the audiological testing session. In contrast, the constant tinnitus group appeared to be more homogenous and reliable during the follow-ups, suggesting that this subgroup of tinnitus may be more reliably identified by means of wave V latency changes. Unlike in any of the previous studies we know of, we analyzed the left and right ears separately, which does not allow us to compare our results with the existing literature (14). As hearing asymmetry affects the spatial percept of tinnitus (57), we argue that this is the correct approach, since clinicians may also want to evaluate other forms of tinnitus (e.g., unilateral) and make predictions based on responses from specific brain stem structures or pathways.

Our findings also have high clinical relevance, since ABRs can be easily implemented in the laboratory and in the clinic, thus representing a practical and potentially useful objective measure for assessing neural dysfunction. Whether absolute ABR wave V latencies may be used as a metric to objectively identify constant tinnitus on an individual patient basis remains to be determined. Our study reveals that absolute metrics differ depending on the clinical system used in addition to the overlapping contributions of sex, reduced hearing sensitivity, and hyperacusis. One solution to circumventing this issue is to integrate a machine learning and/or artificial intelligence approach that incorporates all essential variables to provide a more optimal “predictive” value, that asks, in other words, “What is the likelihood of a given patient having constant tinnitus?” Future studies will have to resolve how to best parse out such complexities in order to facilitate future clinical trials in determining, for example, whether an anxiolytic improves tinnitus due to an actual change in the neural pathways that generate tinnitus or whether the benefits are solely due to a reduced negative emotional response to tinnitus.

Overall, we show that occasional forms of tinnitus are extremely dynamic, but once they transition to constant tinnitus, they are more likely to remain constant. Constant tinnitus distinguishes itself from occasional tinnitus and nontinnitus with increased wave V latencies of the ABR on the left ear. We propose increased ABR wave V latency as a means of subtyping constant tinnitus. Such a measure could be used to stratify patients during recruitment and potentially as an objective outcome measure whereby improvements in tinnitus could be associated with restored latency values. Our research calls for new definitions of tinnitus that can distinguish individuals based on neurological and clinical features, as today’s clinical definition of tinnitus is not specific enough.

## Methods

### Longitudinal analysis

#### Study population.

In this part of the study, participants from the longitudinal cohort study SLOSH were used (58). The SLOSH population consists of the participants of the Swedish Work Environment Surveys (SWES) in 2003–2011 (*n* = 40,877). SWES is a subsample of the Labor Force Survey (LFS), representative of the Swedish working population, and is collected in 2-year intervals by Statistics Sweden. Every second year since 2006, the participants in SWES (2003–2011) were invited to respond to the SLOSH postal questionnaires delivered in two versions, one for those who are gainfully employed and one for those who are not gainfully employed or work less than 30% of full time. In this study, all the participants who responded at least twice to any of the two versions of the questionnaire between the 2008 (wave 2) and 2018 (wave 7) data collections were included. All observations with missing values in the question covering experienced tinnitus were removed, leaving a final sample of 20,439 participants with 53,273 observations. The ethnicity of the participants was not available; however, 94% of the SLOSH participants were born in Sweden, most of the remaining (5%) were born elsewhere in Europe, and very few (about 1%) were born outside of Europe.

#### Measures.

The outcome was a binary variable indicating the presence or absence of constant tinnitus (0 = no constant tinnitus; 1 = constant tinnitus). This variable was dichotomized from the following question in the SLOSH questionnaires: “Have you, during the most recent time, experienced sound in any of the ears without there being an external source (so-called tinnitus) lasting more than 5 minutes?” The response alternatives were “no,” “yes, sometimes,” “yes, often,” and “yes, constant.” If the response was “yes, constant,” then the outcome was coded as constant tinnitus; otherwise, the outcome was coded as not-constant tinnitus.

#### Covariates.

The analysis was controlled for age, sex, previous experience of tinnitus, time, and education. Age, sex, and education were collected from the register data linked to the questionnaire data with the Swedish personal identification number. Demographic reporting was defined by the investigators. The age was measured as the participant’s age at the end of the year the survey was answered. The sex was dichotomous (0 = male, 1 = female). The measure of education was categorized into the following groups: primary education or lower (≤9 years), lower secondary education (10–11 years), upper secondary education (12–13 years), first stage of tertiary education (<3 years of tertiary education), and second stage of tertiary education (≥3 years of tertiary education). Previous experience of tinnitus was derived from the response to the tinnitus question (as presented above) at the 1-time lag previous questionnaire (0 = no; 1 = yes, sometimes; 2 = yes, often; 3 = yes, constant), i.e., the experienced tinnitus one wave (2 years) before the outcome was measured. Time was measured as the time when the outcome was measured by year (1 = 2010, 2 = 2012, 3 = 2014, 4 = 2016, 5 = 2018).

#### Data analysis.

GEEs (59) were used to analyze the repeated-measure SLOSH data. Compared with a naive logistic regression, the GEE adjusts for the within-individual correlation, which appears when using longitudinal data, by assuming a working correlation structure (correlation matrix) for the outcome variable a priori. However, no straightforward way exists to know which correlation structure to use, especially not when analyzing a dichotomous outcome variable. Liang and Zeger (1993) argued that GEE analysis is robust against the misspecification of correlation structure, but suggested repeating the analysis with different correlation structures to examine the sensitivity in the parameter estimates (60). For transparency and comparison reasons, 2 extreme correlation structures are presented here apart from the naive logistic regression. The first is an unstructured correlation structure in which all the correlation coefficients are assumed to be different (model 1). The second is an exchangeable structure in which all the correlation coefficients are assumed to be the same (model 2). For comparison reasons, a naive logistic regression model (model 3) is presented as well. In all the models, we analyzed the effect of the previous state of tinnitus (no, sometimes, often, constant) on developing/maintaining constant tinnitus after adjusting for age, sex, education, and time. The procedure Genmod in the statistical software SAS (version 9.4) was used to run all the GEE models.

### Electrophysiological study

Adult participants (>18 years old) were recruited to the STOP through different channels. Some had previously participated in another nationwide general health study (LifeGene; ref. 61) and agreed to be contacted for other studies. The participants were registered on the STOP website (https://stop.ki.se), also open to the public, and some additional recruitment was done through posters and social media advertising the project. After registering, the participants received detailed information and a consent form via mail. Having returned the signed consent forms, they were invited via a secure and personal link to answer the questionnaires on an online platform. The participant flow diagram is reported in Figure 1. Information on ethnicity was not available; however, place of birth could be determined, with the majority of the participants born in Sweden (*n* = 341; 88.5%), others from Europe, the Middle East, or North Africa (*n* = 33; 8.5%), 9 participants designated as other (2.3%), and 2 with designations unknown (0.5%).

### Questionnaires

Between June 2016 and January 2020, 5671 participants responded to the online questionnaires (see below). The questionnaires used were translated into Swedish and validated for online use and have previously been described in detail (27). In short, the online survey consisted of the Tinnitus Sample Case History Questionnaire (TSCHQ), the THI, the Tinnitus Functional Index (TFI), the Tinnitus Catastrophizing Scale (TCS), the Fear of Tinnitus Questionnaire (FTQ), the Hospital Anxiety and Depression Scale (HADS), the Perceived Stress Questionnaire (PSQ-30), the HQ, and 4 domains of the World Health Organization Quality of Life Scale (WHOQoL-BREF). The European School for Interdisciplinary Tinnitus Research (ESIT) screening questionnaire (62) was added to the platform in November 2018 and was answered by 4590 participants (80.9%). Demographic reporting was defined by the investigators.

### Audiological testing

Participants located in the Stockholm area or Skåne who had answered the questionnaire were invited to a battery of auditory measurements at one of three locations. For external validity, the measurements were performed at three different sites: two different hospital audiology clinics in Stockholm (Karolinska Sjukhuset Rosenlund, *n* = 778; and Karolinska Sjukhuset Solna, *n* = 120) and the Department for Logopedics, Phoniatrics, and Audiology at Lund University (Lund, Sweden) (*n* = 29). KS Solna informed the patients about the ongoing study and included interested tinnitus patients as participants. Between August 2016 and December 2019, auditory measurements were collected for 927 participants. The session started with a short interview, confirming the participants’ tinnitus statuses, and otoscopy was performed. The DPOAEs were measured using Madsen Capella 2 (Otometrics) with L1 = 65 dB SPL and L2 = 55 dB SPL at 10 points per octave between F2 = 1 kHz and F2 = 10 kHz. Pure tone audiometry was performed at standard (0.125–8 kHz) and high (8–16 kHz) frequencies using a Madsen Astera 2 clinical audiometer (Otometrics) and HDA 200 headphones (Sennheiser) using a fixed-frequency Bekesy audiometry with a pulsed pure tone (550 ms, 50% duty cycle). Bone conduction thresholds were verified manually for thresholds greater than 20 dB HL between 0.125 and 4 kHz. Speech-in-noise testing was performed using the clinical standard method and material in Sweden, presenting 50 CNC words in +4 dB SNR per ear. The LDLs were assessed at all threshold frequencies. A 1.5-second pure tone was presented at 60 dB HL, increased by 5 dB, and presented again. The subject was instructed to inform the tester if the tone presented would have been uncomfortable to listen to for a longer period (1–2 minutes). The participants with tinnitus were tested with an additional battery of psychoacoustic tinnitus measurements consisting of 2 alternative forced-choice pitch and loudness matching, masking the threshold using one-third-octave narrow band noise, minimal masking level, and residual inhibition. The session concluded with measurements of the ABR, detailed below.

### Measurements of ABR

Two setups for the ABR recordings were used in this study, the Madsen EP200 Chartr (Otometrics) and the Interacoustics Eclipse. The settings for both systems were identical, with high and low pass filters of 0.1 and 3 kHz, respectively, with 100 μs click stimuli of alternating polarity presented at 9.1 clicks/s at 90 dB HL through insert earphones, with contralateral masking of –40 dB relative to the stimulus ear. Each recording consisted of 2000 accepted clicks. The participants were relaxed in a reclined position in a dimly lit room during the recording.

A flowchart of participant recruitment and exclusion is presented in Figure 1, showing a total number of 405 participants. The ABRs were collected on the Chartr for 283 participants and on the Eclipse for 122 participants. For internal validity, the ABRs were collected by 4 licensed audiologists. Initial data collection using the Chartr consisted of 2000 accepted sweeps collected twice per ear. The protocol used with the Eclipse hardware added a condition of 80 dBnHL stimulation at 9.1 clicks/s and 90 dBnHL at 21.2 clicks/s. These conditions were not considered for this analysis. Here, we analyzed the first ABR per ear collected at 90 dBnHL, 9.1 clicks/s. The signal vectors of the collected ABRs were analyzed by a custom MATLAB script that identified relevant ABR features, i.e., wave I, III, and V peaks and troughs. The automatically identified waveform features were then double checked and, if necessary, corrected by 2 independent licensed audiologists blinded to the tinnitus status of the source participant. The test-retest data performed by a single audiologist is shown in Supplemental Table 7.

### Statistics

The sociodemographic variables and questionnaire responses from the participants were summarized for the 3 groups that reported different tinnitus statuses. The within-group proportions are reported as percentages for nominal variables and compared among groups with the χ^2^ test. The numerical variables were compared among all 3 groups with the ANOVA test and for tinnitus-specific questionnaires with 2-tailed Student’s *t* test (arsenal R package).

In investigating whether the ABR is a useful electrophysiological biomarker for tinnitus (occasional or constant), variables known to affect the ABR response will have to be controlled. Other than the ABR variables, our covariates for modeling included age, sex, hearing, hyperacusis, and hardware. Hearing included 2 variables per ear, the clinical standard of 4 frequencies (0.5, 1, 2, 4 kHz), PTA 4, and an average of high-frequency thresholds above 8 kHz (PTA HF). Hyperacusis is included in the questionnaire score from the HQ. A covariate for “hardware” includes information on whether the measurement was performed using the Chartr or Eclipse system. These additional covariates (age, sex, HQ, PTA 4, PTA HF, hardware) were included in a stepwise selection to minimize the BIC evaluating both backward and forward selection. A *P* value of less than 0.05 was considered significant. All of the data analysis was performed using R (R Core Team, 2020) or JMP 14 (SAS Institute Inc.; packages: arsenal, psych).

### Study approval

The project has been approved by the local ethics committee, Regionala etikprövningsnämnden in Stockholm (2015/2129-31/1). Informed consent was obtained from all of the participants prior to participation after clarifying the nature and possible consequences of the study.

## Author contributions

CRC designed and directed the research. NKE and GM carried out the experiments. AL and IU coordinated data collection at the Karolinska Hospital. NKE, GM, JB, and MC analyzed the data and generated the tables. BC, AL, NKE, MC, CL, IU, JB, and CRC discussed the results and wrote the manuscript. All of the authors reviewed the manuscript.

## Supplementary Material

Supplemental data

ICMJE disclosure forms

## Figures and Tables

**Table 1:**
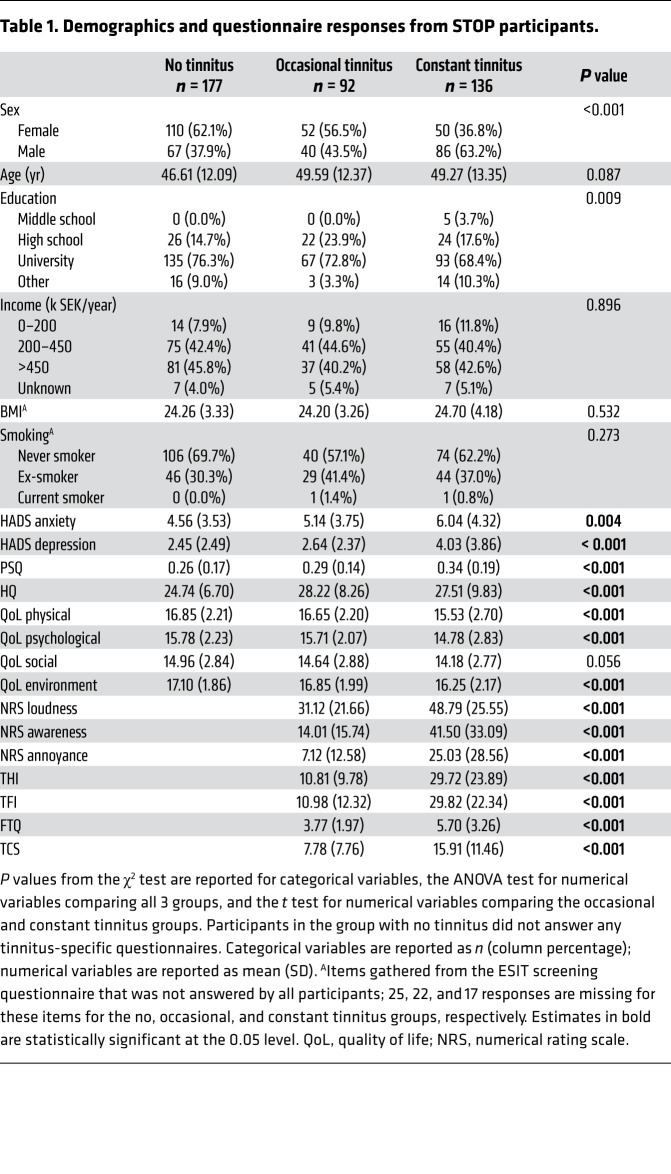
Demographics and questionnaire responses from STOP participants.

**Table 2:**
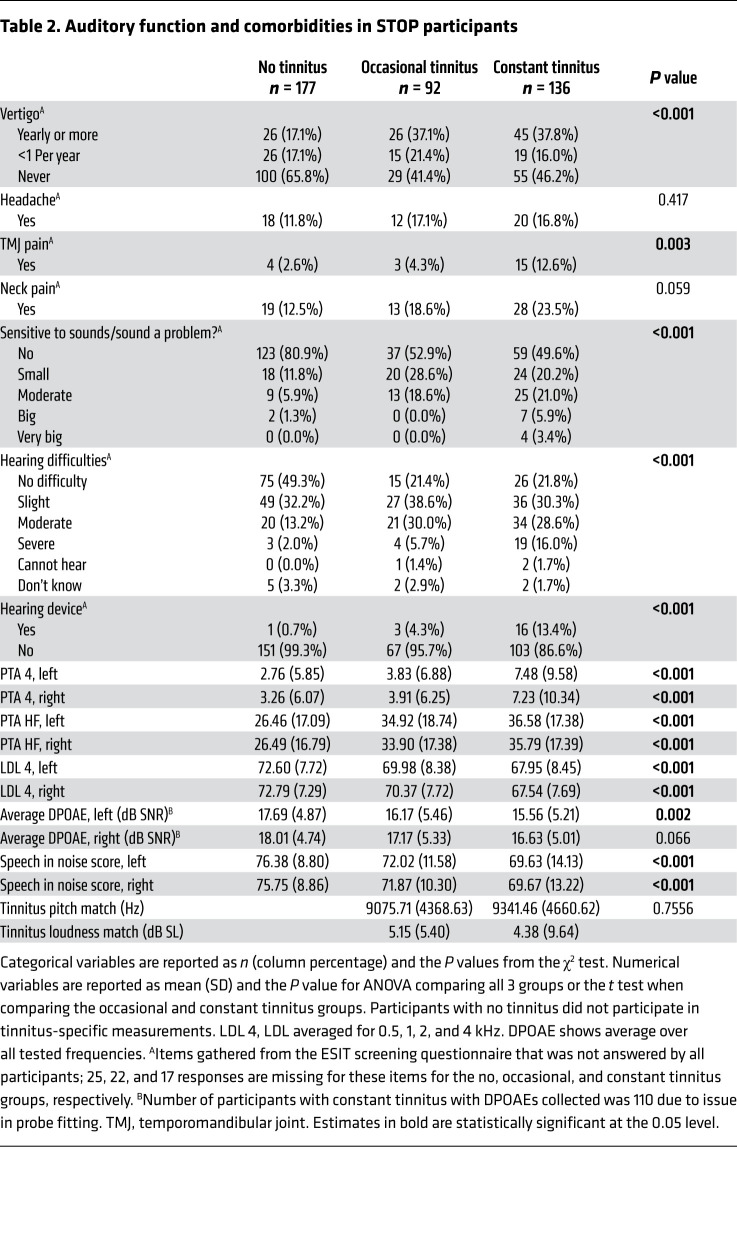
Auditory function and comorbidities in STOP participants

**Table 3:**
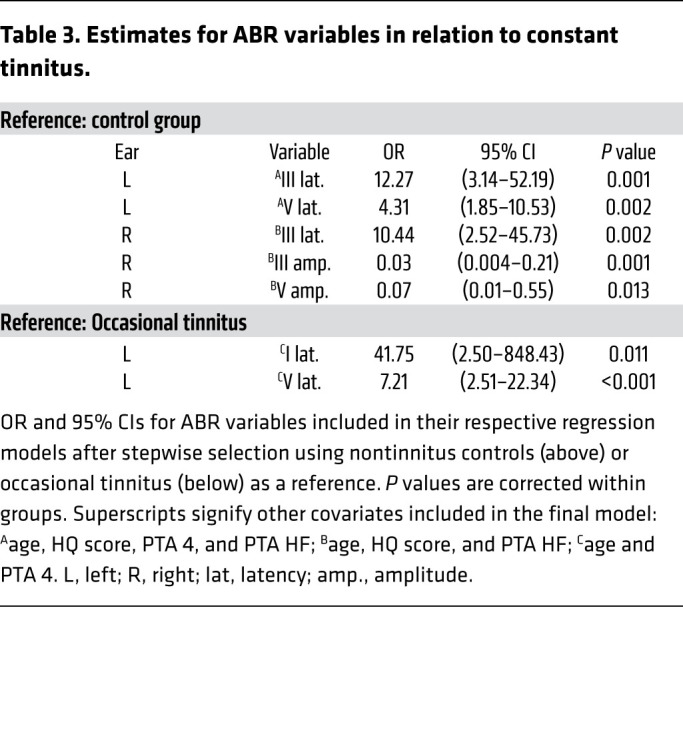
Estimates for ABR variables in relation to constant tinnitus.

## Version 1 01/25/2022

In-Press Preview

## Version 2 03/01/2022

Electronic publication
