# Supplementary material for: Alterations in auditory brain stem response distinguish occasional and constant tinnitus
Source: J Clin Invest. 2022 Mar 1;132(5):e155094. doi: 10.1172/JCI155094 (PMC8884914; doi:10.1172/JCI155094)
Supplement: Supplemental data [file jci-132-155094-s047.pdf]

Supplementary Material:

Univariate regression for constant tinnitus vs no tinnitus

		OR	Low CI	High CI	p corr.
Amplitude	L - wave I	0.06	0.01	0.27	**<0.001**
	L - wave III	0.04	0.01	0.28	**0.002**
	L - wave V	0.12	0.02	0.68	**0.02**
	R - wave I	0.02	0	0.11	**<0.001**
	R - wave III	0.02	0	0.11	**<0.001**
	R - wave V	0.04	0.01	0.27	**0.002**
Latency	L - wave I	30.82	6.71	154.64	**<0.001**
	L - wave III	35.47	10.44	131.61	**<0.001**
	L - wave V	7.69	3.73	16.73	**<0.001**
	L - wave III-I	5.13	1.37	20.69	**0.02**
	L - wave V-I	4.17	1.86	9.67	**<0.001**
	R - wave I	32.39	6.65	173.81	**<0.001**
	R - wave III	20.9	6.3	74.49	**<0.001**
	R - wave V	4.68	2.23	10.21	**<0.001**
	R - wave III-I	3.99	1	16.53	0.054
	R - wave V-I	2.78	1.21	6.52	**0.02**

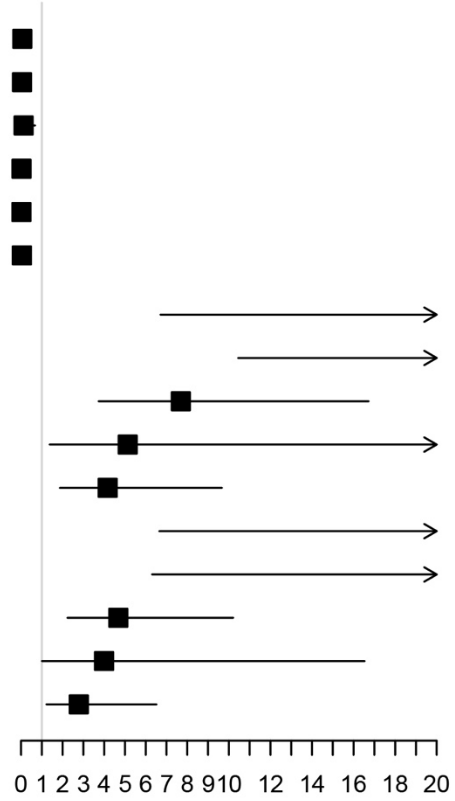

Figure S1. Forest plot of odds ratios and 95% confidence intervals from univariate logistic regression models of auditory brainstem response variables for constant tinnitus using non-tinnitus controls as a reference.

Legend: Arrows indicate an odds ratio and/or upper confidence interval > 20. L: left ear, R: right ear, OR: odds ratio, CI: confidence interval, p corr.: p-value corrected with the method of Benjamini & Hochberg, significant findings ($p < 0.05$) in bold.

Univariate regression for occasional tinnitus vs no tinnitus

		OR	Low CI	High CI	p corr.
Amplitude	L - wave I	0.09	0.01	0.49	**0.014**
	L - wave III	0.25	0.04	1.48	0.192
	L - wave V	0.26	0.04	1.82	0.233
	R - wave I	0.03	0	0.19	**0.002**
	R - wave III	0.03	0	0.23	**0.004**
	R - wave V	0.02	0	0.19	**0.004**
Latency	L - wave I	7.86	1.59	41.11	**0.023**
	L - wave III	8.15	2.29	30.93	**0.005**
	L - wave V	2.2	1.09	4.58	0.052
	L - wave III-I	2.43	0.59	10.6	0.27
	L - wave V-I	1.34	0.6	3.03	0.476
	R - wave I	16.65	2.74	111.05	**0.007**
	R - wave III	7.5	2.02	29.28	**0.007**
	R - wave V	3.51	1.58	8.11	**0.007**
	R - wave III-I	2.4	0.48	12.18	0.325
	R - wave V-I	2.8	1.1	7.37	0.053

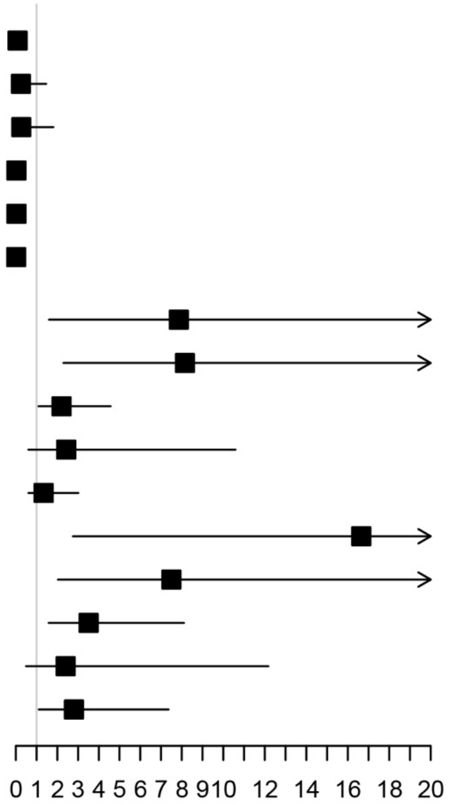

Figure S2. Forest plot of odds ratios and 95% confidence intervals from univariate logistic regression models of auditory brainstem response variables for occasional tinnitus using non-tinnitus controls as a reference.

Legend: Arrows indicate an upper confidence interval > 20. L: left ear, R: right ear, OR: odds ratio, CI: confidence interval, p corr.: p-value corrected with the method of Benjamini & Hochberg, significant findings ($p < 0.05$) in bold.

Univariate regression for constant tinnitus vs occasional tinnitus

		OR	Low CI	High CI	p corr.
Amplitude	L - wave I	0.63	0.1	3.88	0.73
	L - wave III	0.2	0.02	2.05	0.427
	L - wave V	0.43	0.05	3.5	0.686
	R - wave I	0.55	0.08	3.68	0.73
	R - wave III	0.53	0.05	5.41	0.73
	R - wave V	1.15	0.12	11.38	0.94
Latency	L - wave I	3.92	0.59	27.37	0.427
	L - wave III	4.34	1.05	18.87	0.156
	L - wave V	3.17	1.38	7.55	0.051
	L - wave III-I	2.08	0.4	11.29	0.686
	L - wave V-I	3.3	1.21	9.41	0.106
	R - wave I	2.32	0.39	14.22	0.686
	R - wave III	3.27	0.78	14.23	0.324
	R - wave V	1.23	0.51	2.98	0.73
	R - wave III-I	1.66	0.36	7.86	0.73
	R - wave V-I	1.02	0.39	2.68	0.963

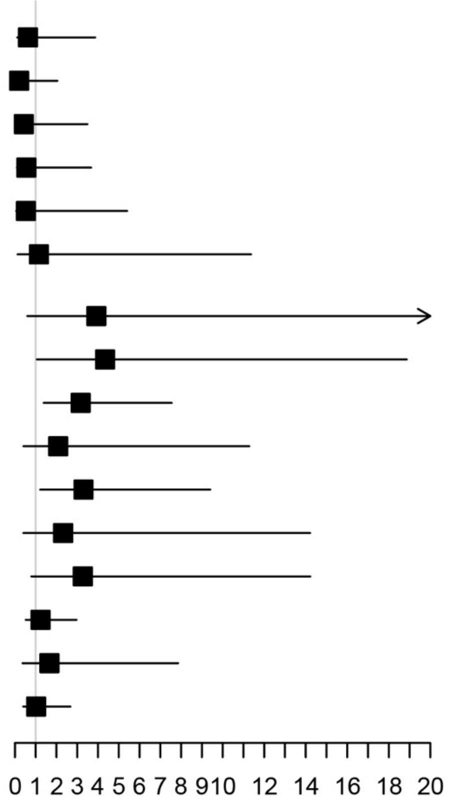

Figure S3. Forest plot of odds ratios and 95% confidence intervals from univariate logistic regression models of auditory brainstem response variables for constant tinnitus using occasional tinnitus as a reference.

Legend: The arrow indicates an upper confidence interval > 20. L: left ear, R: right ear, OR: odds ratio, CI: confidence interval, p corr.: p-value corrected with the method of Benjamini & Hochberg.

	Wave 3 (2010) (n=8,505)		Wave 4 (2012) (n=7,849)		Wave 5 (2014) (n=7,363)		Wave 6 (2016) (n=15,271)		Wave 7 (2018) (n=14,285)	
	Occ	Const	Occ	Const	Occ	Const	Occ	Const	Occ	Const
Previous wave tinnitus state, n (%)										
No tinnitus	6,142 (72.2)	33 (0.4)	5,608 (71,5)	45 (0,6)	5,257 (71.5)	59 (0.8)	10,421 (68.2)	102 (0.7)	9,533 (66.7)	98 (0.7)
Some tinnitus	1,250 (14.7)	50 (0.6)	1,158 (14.8)	48 (0.6)	1,049 (14.3)	72 (1.0)	2,408 (15.8)	122 (0.8)	2,183 (15.3)	135 (1.0)
Often tinnitus	314 (3.7)	69 (0.8)	312 (4.0)	81 (1.0)	250 (3.4)	85 (1.2)	609 (4.0)	206 (1.4)	669 (4.7)	203 (1.4)
Constant tinnitus	166 (2.0)	481 (5.7)	146 (1.9)	451 (5.8)	98 (1.3)	493 (6.7)	275 (1.8)	1,128 (7.4)	278 (2.0)	1,186 (8.3)
Gender, n (%)										
Male	3,267 (41.5)	401 (63.4)	3,002 (41.6)	401 (64.2)	2,720 (40.9)	446 (62.9)	5,598 (40.8)	928 (59.6)	5,146 (40.6)	936 (57.7)
Female	4,605 (58.5)	232 (36.7)	4,222 (58.4)	224 (35.8)	3,934 (59.1)	263 (37.1)	8,115 (59.2)	630 (40.4)	7,517 (59.4)	686 (42.3)
Age										
mean (sd)	52.0 (11.3)	56.4 (10.0)	54.3 (11.2)	58.2 (9.8)	56.2 (11.1)	60.2 (10.1)	56.1 (11.4)	59.6 (9.9)	58.0 (11.4)	61.3 (9.8)
range	22 - 72	22 - 71	24 - 74	24 - 73	27 - 76	26 - 75	21 - 78	24 - 77	23 - 80	26 - 79
Education, n (%)										
Primary education or lower	1,227 (15.6)	115 (18.2)	1,126 (15.6)	106 (17.0)	979 (14.7)	116 (16.4)	1,731 (12.6)	221 (14.2)	1,504 (11.9)	237 (14.6)
Lower secondary education	1,772 (22.5)	163 (25.8)	1,579 (21.9)	155 (24.8)	1,430 (21.5)	174 (24.5)	2,763 (20.2)	370 (23.8)	2,509 (19.8)	377 (23.2)
Upper secondary education	1,757 (22.3)	129 (20.4)	1,586 (22.0)	131 (21.0)	1,456 (21.9)	150 (21.2)	3,015 (22.0)	353 (22.7)	2,724 (21.5)	378 (23.3)
First stage of tertiary education	1,097 (13.9)	80 (12.6)	1,018 (14.1)	77 (12.3)	940 (14.1)	96 (13.5)	1,958 (14.3)	198 (12.7)	1,861 (14.7)	209 (12.9)
Second stage of tertiary education	2,017 (25.6)	146 (23.0)	1,914 (26.5)	156 (25.0)	1,849 (27.8)	173 (24.4)	4,244 (31.0)	416 (26.7)	4,060 (32.1)	421 (26.0)

Table S1. Characteristics of the SLOSH participants in wave 3 (2010) – wave 7 (2018)

‘

Flow	Frequency	Percent	Cumulative frequency	Cumulative percent
**00**	33231	62.38	33231	62.38
**01**	3317	6.23	36548	68.61
**02**	413	0.78	36961	69.38
**03**	337	0.63	37298	70.01
**10**	2998	5.63	40296	75.64
**11**	4096	7.69	44392	83.33
**12**	954	1.79	45346	85.12
**13**	427	0.80	45773	85.92
**20**	265	0.50	46038	86.42
**21**	862	1.62	46900	88.04
**22**	1027	1.93	47927	89.96
**23**	644	1.21	48571	91.17
**30**	125	0.23	48696	91.41
**31**	266	0.50	48962	91.91
**32**	572	1.07	49534	92.98
**33**	3739	7.02	53273	100.00

Table S2. Chart measuring the flow between the tinnitus states for all 53,273 observations in the SLOSH study.

Legend: The first digit in the column “Flow” measuring the previous state of tinnitus and the second digit measuring the two year follow up tinnitus state. 0 = no tinnitus, 1 = sometimes tinnitus, 2 = often tinnitus and 3 = constant tinnitus. Hence, “00” measures those who first answered no tinnitus in two waves in a row. “03” measures those who first answered no tinnitus and then constant tinnitus in the next wave

Parameter	Estimate (SE)	Z	P-value	OR	95 % CI	CL range
Model 1 - Unstructured correlation structure						
Previous tinnitus (sometimes)	1.727 (0.078)	22.22	<.0001	5.62	4.83-6.55	1.72
Previous tinnitus (often)	3.392 (0.075)	45.48	<.0001	29.74	25.69-34.42	8.73
Previous tinnitus (constant)	6.402 (0.071)	90.24	<.0001	603.02	524.74-692.98	168.24
Model 2 - Exchangeable correlation structure						
Previous tinnitus (sometimes)	1.755 (0.077)	22.68	<.0001	5.78	4.97-6.73	1.76
Previous tinnitus (often)	3.474 (0.074)	47.05	<.0001	32.25	27.91-37.27	9.36
Previous tinnitus (constant)	6.2873 (0.073)	86.37	<.0001	537.72	466.22-620.18	153.96
**Parameter**	**Estimate (SE)**	***Chi-square***	**P-value**	**OR**	**95 % CI**	**CL range**
Model 3 - Naïve logistic						
Previous tinnitus (sometimes)	1.735 (0.074)	548.68	<.0001	5.67	4.90-6.55	1.65
Previous tinnitus (often)	3.435 (0.072)	2297.20	<.0001	31.01	26.95-35.69	8.74
Previous tinnitus (constant)	6.001 (0.067)	8132.93	<.0001	403.91	354.52-460.17	105.65

Table S3. Results of the generalized estimation equations (GEE) models measuring the effect of previous state of tinnitus on developing/maintain constant tinnitus in SLOSH.

Legend: All models are adjusted for age, gender, time and education

Parameter	Estimate (SE)	Z	P-value	OR	95 % CI	CL range
Model 1 - Unstructured						
Previous tinnitus (sometimes)	1.665 (0.111)	15.01	<.0001	5.29	4.25-6.57	2.32
Previous tinnitus (often)	3.301 (0.105)	31.57	<.0001	27.14	22.11-33.31	11.20
Previous tinnitus (constant)	6.401 (0.100)	64.19	<.0001	602.17	495.27-732.14	236.87
Model 2 - Exchangeable						
Previous tinnitus (sometimes)	1.693 (0.111)	15.22	<.0001	5.44	4.37-6.76	2.39
Previous tinnitus (often)	3.401 (0.104)	32.68	<.0001	30.01	24.47-36.80	12.33
Previous tinnitus (constant)	6.299 (0.103)	61.48	<.0001	544.18	445.17-665.22	220.05
**Parameter**	**Estimate (SE)**	**Chi-square**	**P-value**	**OR**	**95 % CI**	**CL range**
Model 3 - Naïve logistic						
Previous tinnitus (sometimes)	1.676 (0.104)	260.51	<.0001	5.34	4.36-6.55	2.19
Previous tinnitus (often)	3.374 (0.099)	1162.08	<.0001	29.19	24.04-35.44	11.40
Previous tinnitus (constant)	5.895 (0.092)	4071.94	<.0001	363.25	303.09-435.35	132.26

Table S4. Results of the generalized estimation equations (GEE) models measuring the effect of previous state of tinnitus on developing/maintain constant tinnitus only analyzing males in SLOSH.

Legend: All models are adjusted for age, gender, time and education

Parameter	Estimate (SE)	Z	P-value	OR	95 % CI	CL range
Model 1 - Unstructured						
Previous tinnitus (sometimes)	1.772 (0.109)	16.24	<.0001	5.88	4.75-7.28	2.53
Previous tinnitus (often)	3.455 (0.106)	32.52	<.0001	31.67	25.71-39.00	13.29
Previous tinnitus (constant)	6.400 (0.101)	63.24	<.0001	601.95	493.64-734.02	240.38
Model 2 - Exchangeable						
Previous tinnitus (sometimes)	1.798 (0.108)	16.60	<.0001	6.04	4.88-7.47	2.59
Previous tinnitus (often)	3.518 (0.105)	33.43	<.0001	33.70	27.42-41.42	14.00
Previous tinnitus (constant)	6.294 (0.104)	60.82	<.0001	541.55	442.12-663.33	221.21
**Parameter**	**Estimate (SE)**	**Chi-square**	**P-value**	**OR**	**95 % CI**	**CL range**
Model 3 - Naïve logistic						
Previous tinnitus (sometimes)	1.785 (0.106)	285.07	<.0001	5.96	4.84-7.33	2.49
Previous tinnitus (often)	3.485 (0.104)	1115.65	<.0001	32.63	26.59-40.03	13.44
Previous tinnitus (constant)	6.115 (0.096)	4036.66	<.0001	452.44	374.66-546.36	171.70

Table S5. Results of the generalized estimation equations (GEE) models measuring the effect of previous state of tinnitus on developing/maintain constant tinnitus only analyzing females in SLOSH.

Legend: All models are adjusted for age, gender, time and education

		Chartr				Eclipse			
		No tinnitus n=100	Occasional tinnitus n=80	Constant tinnitus n=103	*p*-value	No tinnitus n=77	Occasional tinnitus n=12	Constant tinnitus n=33	*p*-value
	Left ear								
Amplitude (µ V)	Wave I	0.35 (0.15)	0.31 (0.13)	0.32 (0.15)	0.159	0.44 (0.16)	0.46 (0.21)	0.32 (0.16)	**0.003**
	Wave III	0.20 (0.10)	0.20 (0.10)	0.19 (0.09)	0.773	0.35 (0.17)	0.45 (0.15)	0.29 (0.12)	**0.017**
	Wave V	0.35 (0.14)	0.32 (0.13)	0.31 (0.13)	0.051	0.35 (0.14)	0.36 (0.11)	0.32 (0.14)	0.652
Latency (ms)	Wave I	1.48 (0.13)	1.48 (0.13)	1.51 (0.12)	0.261	1.25 (0.12)	1.20 (0.08)	1.34 (0.14)	**0.001**
	Wave III	3.67 (0.19)	3.72 (0.17)	3.77 (0.20)	**0.003**	3.46 (0.19)	3.41 (0.20)	3.60 (0.16)	**0.001**
	Wave V	5.49 (0.33)	5.53 (0.34)	5.67 (0.28)	**<0.001**	5.18 (0.36)	5.04 (0.40)	5.38 (0.30)	**0.008**
	Wave I-III	2.19 (0.21)	2.24 (0.18)	2.25 (0.18)	0.123	2.21 (0.18)	2.21 (0.21)	2.25 (0.16)	0.512
	Wave I-V	4.00 (0.32)	4.04 (0.32)	4.14 (0.25)	**0.007**	3.94 (0.34)	3.84 (0.37)	4.00 (0.24)	0.381
	Right ear								
Amplitude (µ V)	Wave I	0.35 (0.15)	0.29 (0.12)	0.29 (0.15)	**0.006**	0.43 (0.18)	0.42 (0.23)	0.30 (0.16)	**0.005**
	Wave III	0.20 (0.10)	0.19 (0.10)	0.19 (0.10)	0.656	0.40 (0.15)	0.40 (0.13)	0.28 (0.15)	**0.002**
	Wave V	0.34 (0.12)	0.30 (0.10)	0.29 (0.13)	**0.012**	0.37 (0.12)	0.34 (0.12)	0.33 (0.14)	0.325
Latency (ms)	Wave I	1.47 (0.10)	1.48 (0.13)	1.49 (0.15)	0.654	1.27 (0.12)	1.21 (0.12)	1.39 (0.17)	**<0.001**
	Wave III	3.69 (0.17)	3.70 (0.17)	3.75 (0.18)	0.093	3.45 (0.17)	3.43 (0.19)	3.61 (0.21)	**<0.001**
	Wave V	5.51 (0.31)	5.55 (0.29)	5.59 (0.28)	0.181	5.17 (0.28)	5.16 (0.46)	5.33 (0.31)	0.06
	Wave I-III	2.22 (0.16)	2.22 (0.18)	2.25 (0.17)	0.406	2.17 (0.15)	2.22 (0.13)	2.20 (0.24)	0.645
	Wave I-V	4.03 (0.28)	4.07 (0.27)	4.09 (0.27)	0.296	3.90 (0.28)	3.97 (0.43)	3.95 (0.37)	0.721

Table S6. Amplitude and latency of auditory brainstem responses Waves in STOP participants.

Legend: Mean amplitude (in µV) and latency (in ms) values for the investigated ABR parameters stratified by the two hardwares used. Standard deviation are shown in parenthesis. Reported *p*-values are from ANOVA comparing No, Occasional and Constant tinnitus. Estimates in bold are statistically significant at 0.05 level.

		Chartr		Eclipse	
		ICC3	95% CI	ICC3	95% CI
Latency	Wave I	0.90	0.55 - 0.90	0.87	0.71 - 0.94
	Wave III	0.96	0.81 - 0.96	0.92	0.81 - 0.97
	Wave V	0.89	0.49 - 0.89	0.79	0.57 - 0.91
	Wave III-I	0.92	0.82 - 0.97	0.87	0.70 - 0.94
	Wave V-I	0.77	0.52 - 0.90	0.82	0.62 - 0.92
Amplitude	Wave I	0.43	0.02 - 0.71	0.96	0.91 - 0.98
	Wave III	0.26	-0.17 - 0.61	0.18	-0.25 - 0.55
	Wave V	0.47	0.07 - 0.74	0.48	0.08 - 0.74

Table S7. Test-retest reliability of the two ABR systems.

Legend: To compare the two ABR systems used we performed a test-retest for both systems on eight (50% men) young (mean age 31.6 years, SD: 6.5) healthy participants. This group did not complete the full test battery but were tested using high frequency audiometry and the 9.1 clicks/s at 90 dBnHL ABR condition, 2000 clicks, once per ear, for both systems at two sessions around one week apart. Average (± SD) thresholds were within normal ranges with PTA4 of 2.0 (± 8.8) and 2.3 (± 9.7) dB HL, for left and right ears respectively and PTAHF of 15.4 (± 19.26) and 17.1 (± 22.7) dB HL. Latency and amplitude values for ABR waves I, III and V were manually extracted and used to calculate the intraclass correlation coefficient ICC3. The intraclass correlation (ICC3) in a test-retest of the two ABR systems used for this study showed good to excellent reliability (ICC3 > 0.75) for both systems latency measurements. Amplitude measurements generally had poor reliability (ICC3 < 0.5) except for wave I amplitude as measured by the Eclipse system, which showed excellent reliability with ICC3 of 0.96. Reported here are Intraclass correlation coefficients (ICC3) and 95% confidence interval for the studied variables in a test-retest of the two systems used for ABR measurements. Data represents 16 ears from a total of 8 participants.
